# Changes in oral home care and smoking habits during COVID‐19 pandemic: A cross‐sectional study

**DOI:** 10.1002/cre2.840

**Published:** 2024-02-06

**Authors:** Momen A. Atieh, Afrah Aldhanhani, Maanas Shah, Andrew Tawse‐Smith, Nabeel H. M. Alsabeeha

**Affiliations:** ^1^ Department of Periodontology, Hamdan Bin Mohammed College of Dental Medicine, Mohammed Bin Rashid University of Medicine and Health Sciences Dubai Healthcare City Dubai United Arab Emirates; ^2^ Department of Periodontology, Faculty of Dentistry, Sir John Walsh Research Institute University of Otago Dunedin New Zealand; ^3^ Department of Periodontology, School of Dentistry University of Jordan Amman Jordan; ^4^ Department of Dental Services Emirates Health Services Dubai United Arab Emirates

**Keywords:** COVID‐19, dental care, dental health, oral health

## Abstract

**Objectives:**

The coronavirus disease‐19 (COVID‐19) pandemic has caused disruption in the health behavior in many aspects of life. While hand hygiene was promoted as one of the precautionary measures to mitigate and contain COVID‐19, oral health and smoking might have received less attention in the media campaigns. The aim of this study was to examine health behavioral changes in terms of oral home care habits, smoking, and perception of dental care during the COVID‐19 pandemic.

**Material and Methods:**

An online survey was designed to assess oral home care, smoking habits, and attitude toward dental services of participants aged 18 years and older. The data were collected between September and November 2021. The strength of association between changes in oral home care habits, smoking, and attitude toward invasive/long dental procedures and each variable was measured by *χ* ^2^ analysis. Estimates of relative risk were also calculated for all variables. Predictors of avoiding dental procedures were estimated by a binary logistic regression.

**Results:**

A total of 532 participants, based in the United Arab Emirates, took part in this online survey with a response rate of 88.7%. The age of the participants ranged between 18 and 67 with mean age of 34.9 ± 9.0 years. The majority of the participants have adopted changes in their routine oral home care habits, with 82.1% of them changing the toothbrush more frequently. Participants who changed their oral home care habits were more likely to have received sufficient information on the importance of maintaining oral health. Likewise, the changes in smoking habits were significantly associated with receiving information on the relationship between smoking and the severity of the COVID‐19 (*p* < 0.001).

**Conclusions:**

The findings showed that positive behavior toward oral home care and smoking was noticed during the pandemic particularly when public receives sufficient and up‐to‐date information.

## 1 INTRODUCTION

The World Health Organization (WHO) declared coronavirus disease‐19 (COVID‐19) as a pandemic and emphasized the critical importance of containing the virus and preventing community transmission. The most commonly reported routes of transmission for severe acute respiratory syndrome coronavirus 2 (SARS‐CoV‐2) are by inhalation of respiratory droplets from infected individuals that may occur within 1‐m radius or through direct interaction of virus‐infested particles by touching surfaces contaminated with infected respiratory droplets (Chan et al., 2020; Liu, Liao, et al., 2020; Ong et al., 2020). Moreover, SARS‐CoV‐2 has also been detected in the saliva of infected individuals (Atieh et al., 2022; Azzi et al., 2020; To et al., 2020). While opinions vary as to how long SARS‐CoV‐2 survives in aerosol particles or saliva of infected individuals (Van Doremalen et al., 2020; Ong et al., 2020), a consensus seems to exist on the importance of effective hand washing in preventing cross‐infection and reducing the spread of SARS‐CoV‐2 (Alzyood et al., 2020). Mass media campaigns were extensively used to increase public awareness on the importance of hand washing and propagating it as a primary measure in combating the spread of the SARS‐CoV‐2 diseases (Liu, Xie, et al., 2020). Oral home care promotion, on the other hand, received little attention despite that SARS‐CoV‐2 was detected in the saliva of infected individuals even in the presence of negative pharyngeal swabs (Azzi et al., 2020). In fact, a recent report (Eduardo et al., 2022) has shown that the use of a common oral home care measure such as brushing with a toothpaste containing antimicrobial properties could reduce the SARS‐CoV‐2 viral load in saliva by twofold to fourfold. Encouraging results on reducing SARS‐CoV‐2 viral overload was also observed with the home use of mouth rinses (Eduardo et al., 2021).

Smoking is a lifestyle habit that is associated with serious negative effects on the general health and wellbeing of affected individuals (Ambrose & Barua, 2004; Yanbaeva et al., 2007). The immune and pulmonary systems are primarily affected making smokers more susceptible to infections and respiratory diseases (Robbins et al., 2004). A WHO report published in 2020 has shown that smokers were more likely to contract COVID‐19 than nonsmokers, and those already infected with lung disease might worsen their disease condition (WHO, 2020). Moreover, smokers infected with COVID‐19 had higher mortality rates and were more likely to require mechanical ventilation compared with nonsmokers (Guan et al., 2020; Liu, Tao, et al., 2020). The potential benefits of smoking cessation in reducing COVID‐19 associated mortality, however, has rarely been emphasized in mass media channels.

The scaling down of dental services in the early days of the pandemic have limited public access to proper education on oral home care and smoking cessation measures, as well as safe dental visit practices. During the pandemic, world communities have been subjected to massive information from various sources on a multitude of COVID‐19 precautionary measures. These information have impacted health behavior toward health practices and its perceptions and expectations of healthcare services in the fight against the COVID‐19 pandemic. The extent of changes in behavior toward oral health practices during the COVID‐19 pandemic has been globally investigated. For example, those whose income was affected by COVID‐19 state of emergency in Japan were more reluctant to visit their dentist (Koyama et al., 2022), while an overall reduction in dental visits in Taiwan was mostly related to restrictive health policies and manpower resources (Lee et al., 2021). However, the behavior among other individuals toward and oral health practices in other parts of the world is still not clear. Our research hypothesis is that COVID‐19 pandemic played a role in changing oral home care habits, smoking, and public perception of dental care. The aim of this study was to examine the changes in health behavior in terms of oral home care habits, smoking,and perception of dental care during the COVID‐19 pandemic in the United Arab Emirates (UAE).

## 2 METHODS

### 2.1 Study design

A confirmatory cross‐sectional electronic‐based questionnaire study. The Strengthening the Reporting of Observational Studies in Epidemiology statement (Von Elm et al., 2014) was followed in the design and reporting of the present study.

### 2.2 Participant sample

An online sample size calculator was used to determine the sample size (Raosoft, 2020). The Raosoft calculator was recommended by Hightower and Kelly (2012) for calculating sample size and margins of error in survey data. For assuming 5% margin of error, 95% confidence level, and response distribution of 50%, the minimum sample size was 385 participants. Considering that electronic‐based survey studies are associated with a relatively low response rate (Aitken et al., 2008; Crouch et al., 2011), the survey was sent to 600 participants.

### 2.3 Participant selection

An electronic survey questionnaire was sent to participants, between September and November 2021, by an independent survey software company. The survey company has access to national panels and databases to ensure that subpopulations are underrepresented. Members of the general population in the UAE were randomly selected and invited to participate by clicking on a link to the survey. Information on the research objectives were provided before participants consent to take part in the survey. The independent survey company ensured that the study population was a nationally representative sample composed of Emiratis and residents of UAE, who are aged ≥18 years. The age was selected for the convenience of access to online surveys and only those unable to complete the online survey or provide responses were excluded.

### 2.4 Developing the online questionnaire

The email sent to the participants included information on the research objectives and a link to the survey. Participants were required to complete a two‐part questionnaire (Supporting Information S1: Appendix 1). The first part of the questionnaire was related to participants' sociodemographic characteristics including age, gender, place of residence, ethnic group, educational level, and self‐reported health condition. The second part was related to changes in oral home care practice and smoking, as well as participants' expectations of dental services. The questionnaire consisted of a mixture of open‐ended and Likert‐scale questions. The results of the survey questionnaire were collected over a period of 3 months (September to November 2021) with a reminder email sent to nonresponders within 6 weeks of the first email.

### 2.5 Content validation

Two independent researchers were asked to complete a pilot survey to evaluate the applicability of the questionnaire tool. The survey questionnaire was deemed valid by calculating the content validity index (CVI) on the basis of the researchers' ratings of the survey items' relevance (Lynn, 1986; Polit et al., 2007). As only two experts were involved in reviewing the questionnaire, a cut‐off CVI score of ≥0.80 was considered acceptable (Davis, 1992).

### 2.6 Ethical considerations

Participants were provided clear information that their participation was voluntary. They had to answer the first question of the survey to give their consent on taking part in this research before they can proceed with completing the survey. Ethical approval was obtained from the Institutional Review Board of Mohammed Bin Rashid University of Medicine and Health Sciences (MBRU‐IRB‐2021‐7).

### 2.7 Statistical analysis

Descriptive statistics were used to report participants' characteristics in mean values and standard deviations for continuous variables. Frequencies and percentages were used to summarize categorical variables. In case of multiple completed surveys from the same IP address, only the data from the last completed survey was included in the analysis. The strength of association between changes in oral home care habits, smoking, and attitude toward invasive/long dental procedures and each variable was measured by *χ* ^2^ analysis. Estimates of relative risk were also calculated for all variables. Predictors of avoiding dental procedures were estimated by a binary logistic regression which is the appropriate model for a categorical dichotomous outcome (avoidance of invasive or long dental procedures was coded 0, if invasive or long dental procedure was avoided, and 1 if it was not avoided). A backward stepwise method was selected. All predictor variables which had *p* value of less than 0.05 were entered into the analysis and coded in binary format 0 or 1. Then, at each step, the variable with a significance level equal to or larger than 0.05 was removed, until the final model was obtained. A statistical software (IBM Statistical Package for Social Sciences® for windows, Version 28.0: IBM Corp.) was used to perform statistical analyses.

All personal information was deidentified and securely stored with a study code number. No material, which could personally identify participants, was used in any report on this study. In addition, access to the results was limited to the researchers who only involved in this study.

## 3 RESULTS

Out of the 600 questionnaires sent in September 2021, 532 participants provided informed consent and completed the survey with a response rate of 88.7% (Table 1). The majority of the participants were residents in Abu Dhabi and Dubai and of Asian ethnicity. It is worth mentioning that the UAE consists of seven emirates and the expatriate workforce accounts for 88% of a population of around 10 million according to World Bank (2021). The age of the participants ranged between 18 and 67 with mean age of 34.9 ± 9.0 years. Two age groups were broadly defined to cover young adulthood (18–35 years old) and middle age/older adulthood (>35 years old) (Petry, 2002). In terms of educational background, almost half of the participants completed a bachelor's degree while only 7% completed a postgraduate education. Before the pandemic outbreak, 11% were smokers and 34% of the participants described themselves as regular dental attenders. As for the content validity, the total number of items ranked by the two independent researchers as quite or highly relevant was 18. Therefore, the CVI score was above 0.80 which indicated high agreement on the content validity.

**Table 1:** Demographics and behavioral features of participants.

Variables (categories)	*n* (%)
Gender	
Female	297 (55.8)
Male	235 (44.2)
Age groups (years)	
18–35	306 (57.5)
>35	226 (42.5)
Place of residency	
Abu Dhabi	183 (34.4)
Dubai	223 (41.9)
Sharjah	72 (13.5)
Ajman	21 (3.9)
Fujairah	12 (2.3)
Ras Al Khaimah	18 (3.4)
Umm Al Quwain	3 (0.6)
Ethnicity	
Middle‐Eastern	39 (7.3)
South Asian	70 (13.2)
White/Caucasian	1 (0.2)
Asian	399 (75.0)
African	14 (2.6)
Mixed ethnicity	9 (1.7)
Education level	
Some high school	27 (5.1)
High school	164 (30.8)
Bachelor's degree	280 (52.6)
Master's degree	29 (5.5)
PhD	5 (0.9)
Trade school	27 (5.1)
Self‐rated general health	
Excellent	167 (31.4)
Very good	215 (40.4)
Good	142 (26.7)
Fair	8 (1.5)
Changes in oral home care habits	
Yes, increased the time of brushing teeth	240 (45.1)
Yes, started using dental floss/interdental brushes	46 (8.6)
Yes, started using mouth wash	107 (20.1)
No changes	138 (25.9)
Smoking habits before the coronavirus outbreak	
Active smoker	59 (11.1)
Never smoked	384 (72.2)
Past smoker	89 (16.7)
Regular visitor to dentist and/or hygienist before the coronavirus outbreak	
Yes	183 (34.4)
No	349 (65.6)
Changed number of visits to dentist/hygienist during coronavirus outbreak	
Increasing the number of visits	76 (14.3)
Reducing the number of visits	62 (11.7)
No changes	188 (35.3)
Only visit the dentist when in pain	206 (38.7)

### 3.1 Changes in oral home care habits

Majority of the participants have adopted changes in their routine oral home care habits with 82.1% of them changing the toothbrush more frequently during the pandemic outbreak. In addition, 240 participants (45.1%) increased the time of toothbrushing but only 46 participants (8.6%) started using interdental brushes or dental floss. On the other hand, approximately one in four participants did not adopt any changes in oral home care habits. Participants who changed their oral home care habits were more likely to have received sufficient information on the importance of maintaining oral health during the pandemic as 80.0% of those receiving information on oral health have reported some changes in oral home care habits (*p* < 0.001). Irregular dental attenders before the pandemic outbreak were also more likely to change their oral home care habits (*p* = 0.003). There was, however, no significant association between gender, age, educational background, smoking, and changes in oral home care habits (Table 2).

**Table 2:** Behavioral changes (oral home care habits).

Variables (categories)	*n* (%) changed oral home care habits (95% CI)	*p* Value^a^	Relative risk (95% CI)
Gender			
Female	215 (72.4)	0.370	1.052 (0.952, 1.163)
Male	179 (76.2)		
Age groups (years)			
18–35	230 (75.2)	0.548	1.036 (0.935, 1.148)
>35	164 (72.6)		
Education			
Less than university degree	165 (75.7)	0.484	0.964 (0.871, 1,066)
University degree	229 (72.9)		
Received enough information on the importance of cleaning teeth			
Yes	340 (80.0)	<0.001	1.585 (1.306, 1.924)
No	54 (50.5)		
Smoking habits before the coronavirus outbreak			
Active smoker	46 (78.0)	0.761	NA
Never smoked	282 (73.4)		
Past smoker	66 (74.2)		
Regular visitor to dentist and/or hygienist before the coronavirus outbreak			
Yes	150 (82.0)	0.003	1.172 (1.064, 1.291)
No	244 (69.9)		

Abbreviation: CI, confidence interval.

^a^ *χ* ^2^ test.

### 3.2 Changes in smoking habits

The study included 59 smokers and 89 former smokers while the remaining participants were never‐smokers. A total of 55 participants managed to change their smoking habits during the pandemic outbreak. Nine participants have completely stopped smoking while 46 reduced the number of cigarettes smoked. Three out of the nine participants who stopped smoking used either nicotine replacement therapy or prescribed medication while the remaining six stopped smoking without any assistance. Overall, the changes in smoking habits were significantly associated with receiving information on the relationship between smoking and the severity of the COVID‐19 (*p* < 0.001) as 98.1% of the participants, who stopped smoking or reduced number of smoked cigarettes, received such information. The changes in smoking habits were not associated with other factors, such as age, gender, educational background, or regular dental attendance (Table 3).

**Table 3:** Behavioral changes in smoking habits among smokers (*n* = 59).

Variables (categories)	*n* (%) reduced or stopped smoking (95% CI)	*p* Value^a^	Relative risk (95% CI)
Gender			
Female	11 (91.7)	0.810	1.021 (0.848, 1.230)
Male	44 (93.6)		
Age groups (years)			
18–35	33 (91.7)	0.958	0.958 (0.840, 1.093)
>35	22 (95.7)		
Education			
Less than university degree	21 (91.3)	0.639	1.034 (0.891, 1.201)
University degree	34 (94.4)		
Received enough information on the dangers of smoking and the association between smoking and the severity of the coronavirus disease			
Yes	53 (98.1)	0.001	2.454 (0.838, 7.183)
No	2 (40.0)		
Regular visitor to dentist and/or hygienist			
Yes	15 (93.8)	0.921	1.008 (0.867, 1.172)
No	40 (93.0)		

Abbreviation: CI, confidence interval.

^a^ *χ* ^2^ test.

### 3.3 Changes in perception of dental care

A total of 76 participants increased the number of their visits to dentists or hygienists during the pandemic outbreak while 62 participants reduced these visits. The remaining 394 participants did not change their visit routine or only visited the dentist when in pain. The participants, who avoided invasive or longer dental procedures during the pandemic outbreak, were more likely to be females (*p* < 0.001), aged <36 years old (*p* = 0.007) or sought more information regarding the cross‐infection control in dental practice (*p* < 0.001). There was, however, no significant association between educational background, smoking habits, or regular dental attendance and avoidance of invasive or longer dental procedures (Table 4).

**Table 4:** Behavioral changes (dental services).

Variables (categories)	*n* (%) avoided invasive/longer dental procedures (95% CI)	*p* Value^a^	Relative risk (95% CI)
Gender			
Female	202 (68.0)	<0.001	1.463 (1.181, 1.814)
Male	125 (53.2)		
Age groups (years)			
18–35	173 (56.5)	0.007	1.364 (1.084, 1.716)
>35	154 (68.1)		
Education			
Less than university degree	134 (61.5)	1.000	1.000 (0.872, 1,147)
University degree	193 (61.5)		
Smoking habits before coronavirus outbreak			
Active smoker	30 (50.8)	0.133	NA
Never smoked	245 (63.8)		
Past smoker	52 (58.4)		
Regular visitor to dentist and/or hygienist before coronavirus outbreak			
Yes	120 (65.6)	0.161	1.106 (0.965, 1.267)
No	207 (59.3)		
Sought more information regarding cross‐infection control procedures adopted by dental practice			
Yes	247 (70.4)	<0.001	1.592 (1.334, 1.901)
No	80 (44.2)		

Abbreviation: CI, confidence interval.

^a^ *χ* ^2^ test.

The binary logistic regression showed that females, those who are aged <36 years old and those who sought more information regarding the cross‐infection control in dental practice had a statistically significant association with avoiding invasive or longer dental procedures in the final model. The three variables had low standard errors implying a statistically stable model and did not contain a value of 1.00 representing useful and independent predictor variables. The odds ratios showed that females and those aged <36 years old were 0.6 times more likely to avoid invasive or longer dental procedures while those who sought more information regarding the cross‐infection control in dental practice were almost three times more likely to avoid such procedures. The estimates of the logistic regression model, the adjusted odds ratios for the three variables and their 95% confidence intervals are summarized in Table 5.

**Table 5:** Results of logistic regression analysis.

Predictor variable	Avoiding invasive or longer dental procedures during the pandemic outbreak		
	*B* coefficient (SE)	*p* Value	Odds ratio (95% CI)
Female			
(Yes = 0, No = 1)	−0.57 (0.19)	0.002	0.60 (0.39, 0.82)
Aged < 35 years old			
(Yes = 0, No = 1)	−0.52 (0.19)	0.007	0.60 (0.41, 0.87)
Sought more information regarding the cross‐infection control in dental practice (Yes = 0, No = 1)	1.05 (0.19)	<0.001	2.84 (1.95, 4.15)
Model *χ* ^2^ = 11.29; *df* = 6; *p* = 0.08			

Abbreviation: CI, confidence interval.

## 4 DISCUSSION

The present study evaluated the impact of the COVID‐19 pandemic on health behavior in terms of oral home care routine, smoking, and dental visits. The final sample of 532 participants was considered a good representation of the population of the UAE, particularly in terms of gender and age. Our findings showed that the availability of sufficient information on the importance of oral health and the relationship between smoking and severity of the disease played a significant role in participants taking extra care of their oral health, stopping, or reducing smoking. In terms of the public perception of dental services during the pandemic, those who were females, aged <35 years old and sought more information on cross‐infection protocol were more reluctant to undergo an invasive or longer dental procedure during the pandemic outbreak.

The importance of behavioral changes, such as optimal hand hygiene, was emphasized during COVID‐19 as a mean to control cross‐contamination and reduce the spread of SARA‐CoV‐2 virus (Alzyood et al., 2020). However, the positive effects of reinforcing meticulous oral home care or smoking cessation were not equally addressed in public health media campaigns. It was not until the second year into the pandemic that more attention was given to oral health and the positive effects of good oral hygiene on the overall general health. This was clearly demonstrated in a recent cross‐sectional study (Marouf et al., 2021) where a potential association between the severity of periodontal diseases and risk of COVID‐19 complications was shown.

Overall, majority of the participants in the present study adopted new changes in their oral home care practices particularly those who received sufficient information. Among these changes were the frequent change of toothbrushes and the timing of toothbrushing. This observation was in accordance with those in other studies (Caramida et al., 2022; Keles & Sancakli, 2020) where positive changes in lifestyle and oral home care routine during the pandemic were demonstrated. For example, in two of these studies (Cărămidă et al., 2022; Keles & Sancakli, 2020), the use of manual toothbrushes and dental flossing increased during the pandemic with those adopting such changes being more aware of the relationship between oral health and overall general health. In contrast, other studies (Brondani et al., 2021; Gotler et al., 2022) have shown negative behavior such as decreased timing and frequency of toothbrushing during the pandemic. Stress and anxiety were among other factors contributing to such negative changes. In these latter studies, however, it was not clear whether the participants had any access to information on the importance of oral home care during the lockdown period. Interestingly in the present study, irregular dental attenders were more likely to adopt new oral home care habits compared to regular attenders. It could only be speculated that those irregular attenders were more aware of their poor oral health status due to their erratic dental check‐up behavior (Mullally & Linden, 1994). Therefore, the increased frequency of changing the toothbrushes and the increased time of toothbrushing could be seen as a makeup measure to improve their oral health during such an exceptional time of a pandemic.

Smoking cessation or reducing the frequency of smoking was noted in the present study, although only 11% of the participants were smokers before the pandemic. Similar findings were observed in recent studies on Italian and Romanian populations (Cărămidă et al., 2022; Di Renzo et al., 2020) where smoking was reduced among smokers who accounted for 25% and 30% of the total participants, respectively. On the other hand, more smoking was observed among Polish and British populations (Grogan et al., 2022; Sidor & Rzymski, 2020; Tzu‐Hsuan Chen, 2020) which was related to increased stress and lack of physical activity during the lockdown. Further to this, a recent systematic review (Chun et al., 2022) on changes in smoking behavior during the pandemic showed an overall increase in cigarette smoking that was linked to psychological factors. At the same time, this review revealed that more smokers attempted to quit smoking during the pandemic, which is consistent with the finding of the present study. It should be noted here that majority of those who managed to completely cease or reduce smoking in the present study did not rely on any form of in‐person smoking cessation counseling. In fact, exposure of participants in the present study to sufficient information on the negative effects of smoking resulted in 98% of these participants reducing their smoking habits. The use of remote smoking cessation support in the form of telephone or online applications to help quit smoking during the pandemic, rather than conventional counseling session, have been reported with success in a recent study (Jackson et al., 2021).

It is very difficult to compare with others the observations reported in this study on how the availability of information can influence the health behavior changes with regard to oral home care and smoking during the COVID‐19 pandemic due to the limited data available from other studies. Nevertheless, the findings reported in the present study have shown that public health media on COVID‐19 precautionary measures in the UAE had positive effects on the participants' behavior toward oral home care and smoking.

In terms of the participants' expectations and views on dental services during the pandemic, females, those who are aged <36 years old and those who sought more information regarding the cross‐infection control in dental practice were more likely to avoid invasive or longer dental procedures. Other studies (Cotrin et al., 2020; Gonzalez‐Olmo et al., 2022; Peloso et al., 2020) confirmed the current findings with females being more anxious about contracting COVID‐19, while undergoing certain dental procedures such as tooth extraction and endodontics. However, participants over 40 (Martina et al., 2021) or 60 years (González‐Olmo et al., 2022) were more reluctant to visit a dentist as older participants believed to be more susceptible to contracting COVID‐19. The present study, however, showed that the younger group of participants were less willing to undergo invasive or long dental procedures which was relatively in agreement with a previous study (Cotrin et al., 2020) that reported high levels of anxiety toward dental procedures among young participants. This contradiction, however, might be coincidental and related to the different times of observation between the different studies or the individual perceptions of the precautionary information provided.

One finding of interest in the present study is that participants who sought more information on the cross‐infection protocols in dental practices were more likely to avoid dental services. One explanation could be that this group of participants perceived invasive or long dental procedures as a potential risk for contracting COVID‐19 infection. This is regardless of the information provided on the optimized cross‐infection control measures used and the lack of any convincing evidence that dental procedures contribute to the spread of COVID‐19 infection (Thurzo et al., 2022).

This study attempted to provide an insight into the changes on health behavior toward oral home care practices, smoking habits, and dental service expectations during the COVID‐19 pandemic. Nevertheless, there are several limitations that need to be acknowledged in this study: All the data were self‐reported and social desirability, subjectivity or errors in recalling cannot be excluded. Other confounders such as presence of systemic conditions and income,as well as longitudinal trends were not measured as the data were cross‐sectional, thus only behavior during a very specific timing was captured. The data were collected between September and November 2021 when new COVID‐19 cases dropped to 65 by the end of November 2021. Therefore, the data may not be representative of the behavioral changes at other time points during the pandemic. The study aimed to cover three themes which might be judged by some as three separate themes. However, the themes were within the scope exploring changes in health behavior that are relevant to our dental community The questionnaire had several limitations including the lack of precisely defining “sufficient information” and the use of closed questions. In addition, some questions had the option of selecting “no changes” which might not have allowed participants to report negative trends in behavior. The sample did not present all age groups as all the participants were aged <68 years old. The high response rate among young age groups was more related to the ease of using virtual questionnaire among young and young olds (Kelfve et al., 2020). The overall response rate, however, was notably higher than expected allowing the collection of adequate number of responses to have representative picture of the public attitudes and behavior toward oral health practices. Moreover, the current findings are very valuable as they clearly demonstrated that education and access to information may play an important motivator for improving oral home care and reducing consumption of tobacco.

## 5 CONCLUSION

The current findings showed that positive behavior toward oral home care and smoking was noticed during the pandemic particularly when the public receives sufficient and up‐to‐date information. Females, young participants aged <36 years old and those who sought more information regarding the cross‐infection control were more reluctant to undergo invasive or long dental procedures.

## AUTHOR CONTRIBUTIONS

**Momen A. Atieh**: Concept/design; data collection; data analysis/interpretation; drafting article; critical revision of article; approval of article. **Afrah Aldhanhani**: Data collection; critical revision of article; approval of article. **Maanas Shah**: Critical revision of article; approval of article. **Andrew Tawse‐Smith**: Critical revision of article; approval of article. **Nabeel H. M. Alsabeeha**: Critical revision of article; approval of article.

## CONFLICT OF INTEREST STATEMENT

The authors declare no conflict of interest.

## Supporting information

Supporting information.

## Data Availability

The data that support the findings of this study are available on the request from the corresponding author. The data are not publicly available due to privacy or ethical approval.
