# Supplementary material for: Changes in oral home care and smoking habits during COVID‐19 pandemic: A cross‐sectional study
Source: Clin Exp Dent Res. 2024 Feb 6;10(1):e840. doi: 10.1002/cre2.840 (PMC10847705; doi:10.1002/cre2.840)
Supplement: Supplementary file 1 — Supporting information. [file CRE2-10-e840-s001.docx]

**Appendix I: Online questionnaire**

**Information for the participants:**

We are investigating the perceptions of Emiratis and residents of UAE about any changes in cleaning their teeth, smoking habits and their expectations and experience of dental visits during the coronavirus outbreak.

Your participation in this survey is voluntary. We would appreciate your responses and acknowledge the time you take to do this. All responses will be treated confidentially and data will be anonymized. In other words, it will not be possible to identify anyone from any reports.

**Do you consent to take part in this research?**

Yes

No

**Part I:**

What is your age?

Please enter age in years ……..

What is your gender?

Please select one option

- Male
- Female

Where do you live?

Please select one option

- Abu Dhabi
- Dubai
- Sharjah
- Ajman
- Fujairah
- Ras Al Khaimah
- Umm Al Quwain

What is your ethnic group?

Please select one option

- Middle-Eastern
- South Asian
- White / Caucasian
- Asian
- African
- Hispanic
- Mixed ethnicity

What is the highest level of education you have completed?

Please select one option

- Some high school
- High school
- Bachelor’s Degree
- Master’s Degree
- PhD
- Trade School

How would you describe your health?

Please select one option

- Excellent
- Very good
- Good
- Fair
- Poor

**Part II**

**Oral health**

With the increasing awareness on hand washing and use of sanitizers, do you believe that you received enough information on the importance of cleaning your teeth?

- Yes
- No

Have you changed the habit of cleaning your teeth during the coronavirus outbreak?

- Yes, increased the times of brushing my teeth
- Yes, started using dental floss/interdental brushes
- Yes, started using mouth wash
- No changes

Have you changed your toothbrush more frequently during the coronavirus outbreak?

- Yes
- No

Have you started cleaning your toothbrush after use during the coronavirus outbreak?

- Yes
- No

**Smoking habits**

Were you an active/past smoker before coronavirus outbreak?

- Yes, active smoker
- No, never smoked
- Past smoker

During the coronavirus outbreak, have you read information about the dangers of smoking and the association between smoking and the severity of the coronavirus disease?

- Yes
- No

If you are an active smoker, which one of the following products do you smoke?

- Manufactured cigarettes
- Hand-rolled cigarettes
- Pipes
- Cigars
- Waterpipe tobacco smoking
- Electronic cigarettes

If you are an active smoker, have you changed your smoking habits during the coronavirus outbreak?

- Yes, reduced the number of cigarettes
- Yes, stopped smoking
- No change in the number of cigarettes

If you are an active smoker and managed to stop smoking during coronavirus outbreak, which of the following methods you adopted?

- Use of nicotine patches or any nicotine replacement products
- Smoking counselling session
- Use of medication prescribed by my doctor
- Stopped smoking without assistance

**Dental visit expectation and experience:**

Have you been a regular visitor to the dentist and/or hygienist before coronavirus outbreak? (at least one visit per year)

- Yes
- No

If you are a regular visitor to the dental practice, please enter the number of visits per year: …….

During the coronavirus outbreak, have you considered changing the number of visits to dentist and/or hygienist per year?

- Increasing the number of visits
- Reducing the number of visits
- No changes
- Only visit the dentist when in pain

During the coronavirus outbreak, did you seek more information from your dentist/hygienist regarding the cross-infection control procedures adopted by dental practice

- Yes
- No

During the coronavirus outbreak, have you avoided invasive/longer dental procedures due to fear of cross-infection?

- Yes
- No
